# Monilethrix: One Step More on the Ladder of Cytogenetics

**DOI:** 10.4103/0974-7753.66907

**Published:** 2010

**Authors:** Ashish Singh, S Ambujam, S Srikanth, AN Uma

**Affiliations:** Department of Dermatology, Venereology, Leprology, Mahatma Gandhi Medical College and Research Institute, Pondicherry, India; 1 Department of Anatomy, Mahatma Gandhi Medical College and Research Institute, Pondicherry, India

**Keywords:** Acrocentric association, keratosis pilaris, monilethrix

## Abstract

Monilethrix is one of the hair shaft abnormalities with increased fragility of hair. Here we describe a ten-year-old girl with a history of hair loss and breakage of hair since three months of age, associated with keratosis pilaris along with an abnormal microscopic finding of a hair shaft. A cytogenetic study of the patient showed an unexpectedly high degree of Acrocentric association.

## INTRODUCTION

Monilethrix is an autosomal dominant transmitted disease with very few cases of autosomal recessive inheritance. It is caused by the mutation of one of the three genes encoding the type 2 hair cortex keratin-K81, K83, K86.[1,2]

**Figure 1:**
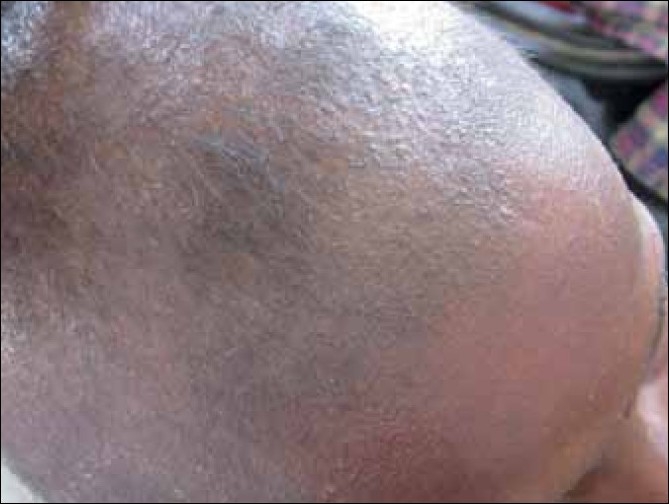
Monilethrix with keratosis pilaris

A ten-year-old girl, born to second degree consanguinous parents, came to our Out Patient Department (OPD), with complaints of loss of hair from the scalp region. Hair on the scalp and elsewhere was normal at birth, but became brittle few weeks after birth. She also developed small papules around the hairs on the scalp [Figure 1]. The birth history of the patient was normal and birth weight was 2.3 kg. Siblings and other family members did not have a similar history.

On examination, we found that hair loss was diffuse, with broken hairs present over the scalp, predominantly involving the occipital region. She also had ciliary and supraciliary madarosis. Examination of the skin showed the presence of goose-flesh-like horny follicular papules mainly involving the occipital scalp, trunk, and extremities. The nails and teeth were found to be normal. An ophthalmological examination was performed, and juvenile cataract was ruled out.

Measurement of weight and height showed growth retardation, however, the girl appeared to be mentally normal. Hemoglobin estimation was completed and iron deficiency anemia ruled out.

**Figure 2:**
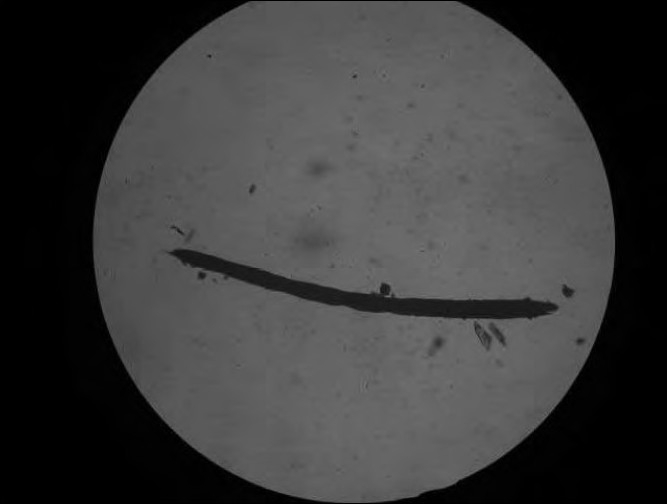
Beaded appearance of hair on microscopy

Microscopic examination of the hairs revealed swollen and narrow bands with beaded hair appearance [Figure 2].

**Figure 3:**
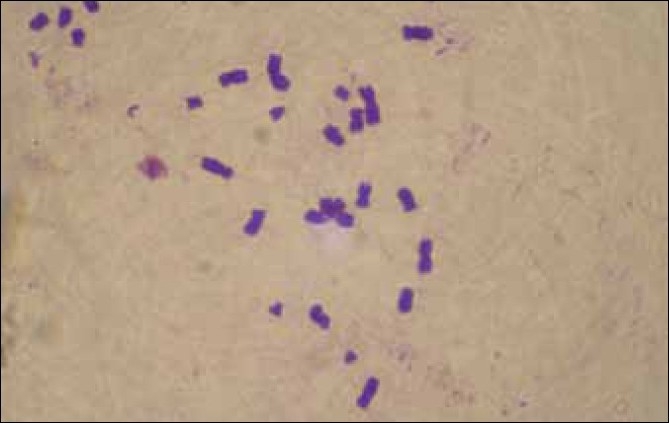
Tetra acrocentric association in the girl

**Figure 4:**
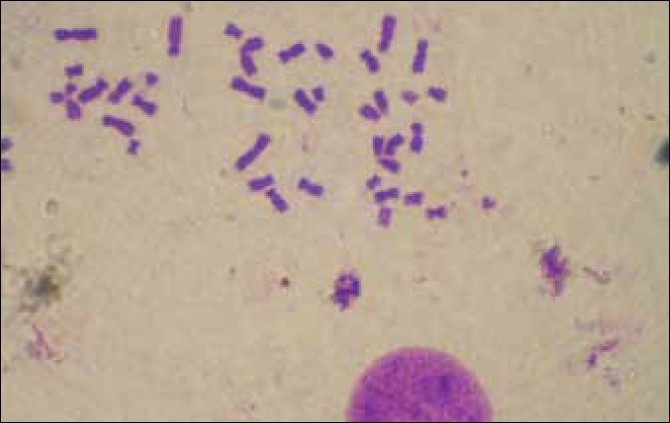
Both Di and Tri acrocentric association in the mother

A cytogenetic study was performed, both on the patient and her mother. Thirty-three metaphases were counted and the results showed 87.87% acrocentric association in the girl [Figure 3] and 57.57% in her mother [Figure 4]. The control of the girl showed 16% acrocentric association, while it was only 27.24% in the four control women. Karyotype of the patient was found to be normal (46xx). Study at the gene level was not possible for us.

## DISCUSSION

Chromosomes are rod-shaped structure with two chromatids joined together at the centromere. Based on the position of the centromere, the chromosomes can be of the Metacentric, Submetacentric, Acrocentric or Telocentric type. In the Acrocentric type, the centromere lies near one end of the chromosome so there are two long arms and two short arms. The tips of these short arms show knob-like structures known as Satellites.

In the Acrocentric Association these Acrocentric Chromosomes, that is, chromosome Nos. 13, 14, 15, 21, and 22 group together at the top of the short arm. The Acrocentric Association may be Di acrocentric, Tri acrocentric, Tetra acrocentric or Penta acrocentric, based on the number of grouped chromosomes (in our patient all the forms were present) and the reason for acrocentric association was the sticky substance formed during nucleolar formation, which had a tendency to hold the associated chromosomes together through mitosis.[3]

Higher degree of Acrocentric Association has been detected in recurrent aborters, oral contraceptive pill (OCP) users, Down’s syndrome, Turner Syndrome (xo), and Klinefelters Syndrome (xxy). These disorders have some impact on chromosomes, and this has been proved in the event of OCP intake and other chromosomal anomalies.[4–8]

The higher incidence of Satellite Association in the patient and her mother could possibly indicate some predisposing factor between the satellite chromosomes and mutation in the microsatellite DNA loci region on chromosome 12, containing the type II Keratin gene cluster. As ours is a single case report, we the authors feel that a large number of case studies are required to establish the relationship between Acrocentric Association and Monilethrix.
